# Electrosurgical Tangential Decortication for Severe Rhinophyma: A Case Report

**DOI:** 10.7759/cureus.109044

**Published:** 2026-05-17

**Authors:** Mario Shuchleib Cukiert, Guillermo Roa Álvarez, Veronica Fonte Ávalos, Judith Dominguez-Cherit, Maria E Vega Memije

**Affiliations:** 1 Dermatology, Hospital General "Dr. Manuel Gea González", Mexico City, MEX; 2 School of Medicine and Medical Sciences, Tecnológico de Monterrey, Mexico City, MEX

**Keywords:** electrocautery, electrosurgery, monopolar electrosurgery, nasal contour reconstruction, nasal remodeling, phymatous rosacea, rhinophyma, rosacea, surgical dermatology

## Abstract

Rhinophyma represents the advanced phymatous stage of rosacea and is characterized by progressive sebaceous hyperplasia, connective tissue overgrowth, and vascular proliferation resulting in nasal deformity. Surgical intervention remains the definitive treatment for rhinophyma once fibrotic hypertrophy is established. Although CO₂ laser therapy has been widely adopted due to precision and hemostasis, electrosurgical tangential decortication remains an effective and economically accessible alternative.

We report the case of a 53-year-old male patient with severe rhinophyma secondary to long-standing rosacea, treated with electrosurgical loop-assisted decortication. Layered excision was performed with preservation of deep pilosebaceous units. Complete re-epithelialization occurred by postoperative day 35 with restoration of nasal contour and no complications. This case underscores that electrosurgery, when performed with meticulous depth control, offers outcomes comparable to laser modalities while maintaining broader accessibility and reduced cost.

## Introduction

Rhinophyma is a progressive, disfiguring manifestation of phymatous rosacea characterized by sebaceous gland hyperplasia, dermal fibrosis, and vascular ectasia [1-3]. Phymatous rosacea represents the most advanced subtype of rosacea and is characterized by progressive tissue hypertrophy and sebaceous gland enlargement, most commonly affecting the nose. It predominantly affects men between the fifth and seventh decades of life and may lead to significant psychosocial distress due to facial disfigurement, social stigma, and the historically inaccurate association with alcohol misuse [4].

The pathophysiology involves chronic inflammation, vascular instability, sebaceous gland hyperplasia, and progressive connective tissue remodeling, ultimately producing thickened skin, enlarged sebaceous glands, and the characteristic bulbous nasal deformity associated with advanced rhinophyma. [5]. Once fibrotic hypertrophy and sebaceous gland enlargement are established, medical therapies are largely ineffective in reversing tissue overgrowth [6].

Surgical management, therefore, becomes the cornerstone of treatment. Available techniques include scalpel excision, dermabrasion, cryosurgery, CO₂ laser ablation, Er:YAG laser resurfacing, and electrosurgical decortication [7-10]. Although these techniques share the common objective of progressive tangential removal of hypertrophic tissue, they differ in energy source, hemostatic control, availability, cost, and degree of thermal injury.

CO₂ laser has gained prominence due to precision and hemostatic control; however, it requires specialized equipment, trained personnel, and significant financial investment [7,8]. Moreover, prolonged erythema and pigmentary alterations have been reported in some series [9].

Electrosurgical tangential decortication, although less frequently highlighted in the recent literature, provides rapid tissue debulking, excellent hemostasis, and wide accessibility. Published series have demonstrated favorable cosmetic outcomes when depth control is meticulously maintained [11-13]. Herein, we present a case of severe rhinophyma treated with electrosurgical decortication and discuss its role as a practical alternative to laser-based techniques.

## Case presentation

**Figure 1:**
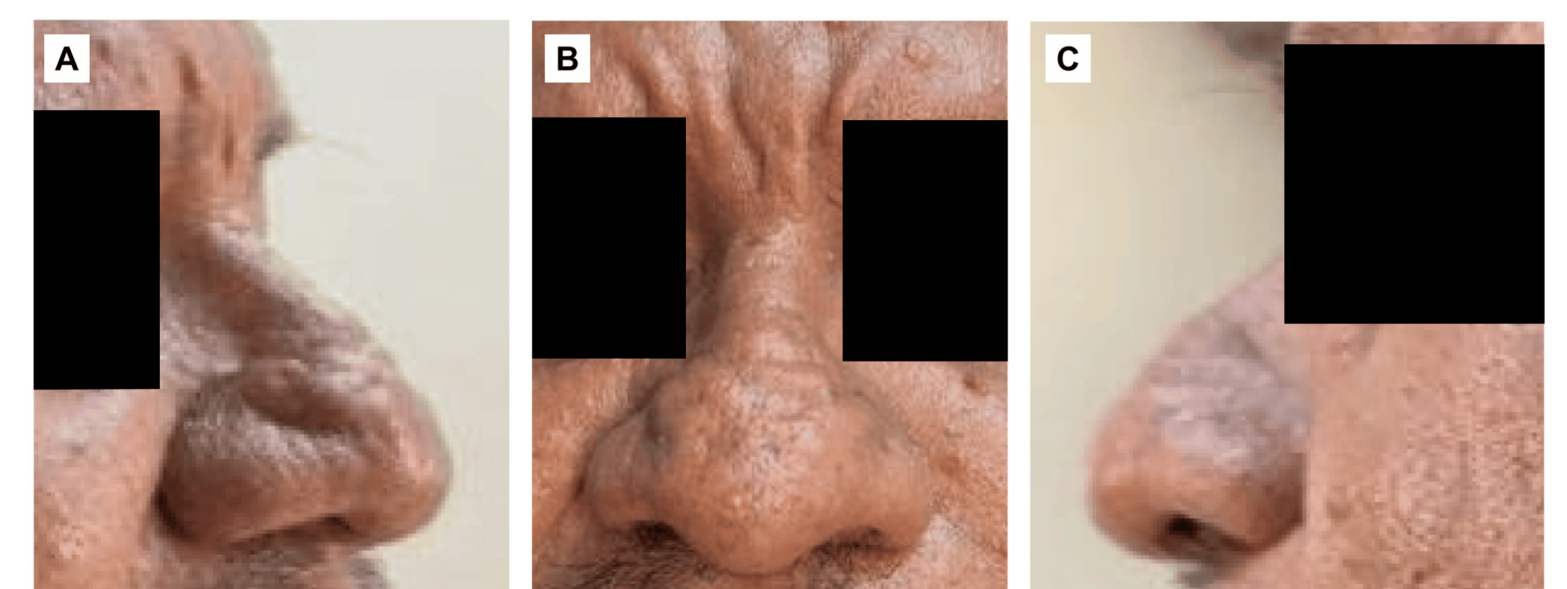
Preoperative clinical presentation: severe rhinophyma (Grade III, lobulated hypertrophy) (A) Left lateral view: Diffuse nodular surface irregularities and sebaceous prominence are observed. The hypertrophy extends across the lower two-thirds of the nose, with preservation of nasal dorsum alignment. (B) Frontal view: Marked nasal hypertrophy is present with an irregular, lobulated contour involving the nasal tip and alae. Prominent follicular openings, sebaceous hyperplasia, and diffuse skin thickening are evident. The overlying skin demonstrates textural irregularity consistent with advanced phymatous change. (C) Right lateral view: Significant projection of the nasal tip is seen with distortion of normal nasal subunit architecture. The alar lobules appear enlarged and indurated, with accentuated telangiectasias.

A 53-year-old man with a more than 10-year history of rosacea presented with progressive nasal enlargement and distortion. He denied systemic comorbidities, prior isotretinoin therapy, or previous surgical procedures. The patient had Fitzpatrick skin phototype III. Nasal enlargement had progressed gradually over several years without periods of rapid acceleration. He denied nasal obstruction, breathing difficulty, or other functional symptoms related to the deformity. Examination revealed severe lobulated hypertrophy of the nasal tip and alae, thickened sebaceous skin with irregular nodularity, dilated follicular ostia, diffuse telangiectasias, and preservation of nasal dorsum alignment. The deformity corresponded to severe (Grade III) rhinophyma (Figures 1A-1C). On palpation, the tissue was diffusely thickened and firm with fibrotic consistency, without focal ulceration or friability.

Differential considerations included basal cell carcinoma arising within rhinophyma and sebaceous carcinoma. No suspicious ulceration, asymmetric destructive change, focal induration, or rapidly enlarging nodules suggestive of malignancy were identified clinically. Histopathologic examination was not performed, which represents a limitation of this report.

Surgical technique

Under local tumescent anesthesia, a loop electrode was used in cutting mode (30-40 W) for tangential decortication. Local anesthesia consisted of approximately 8 mL of 1% lidocaine with epinephrine 1:100,000 administered using tumescent infiltration. Prominent telangiectatic vessels were coagulated prior to tissue debulking to optimize visualization and minimize bleeding.

**Figure 2:**
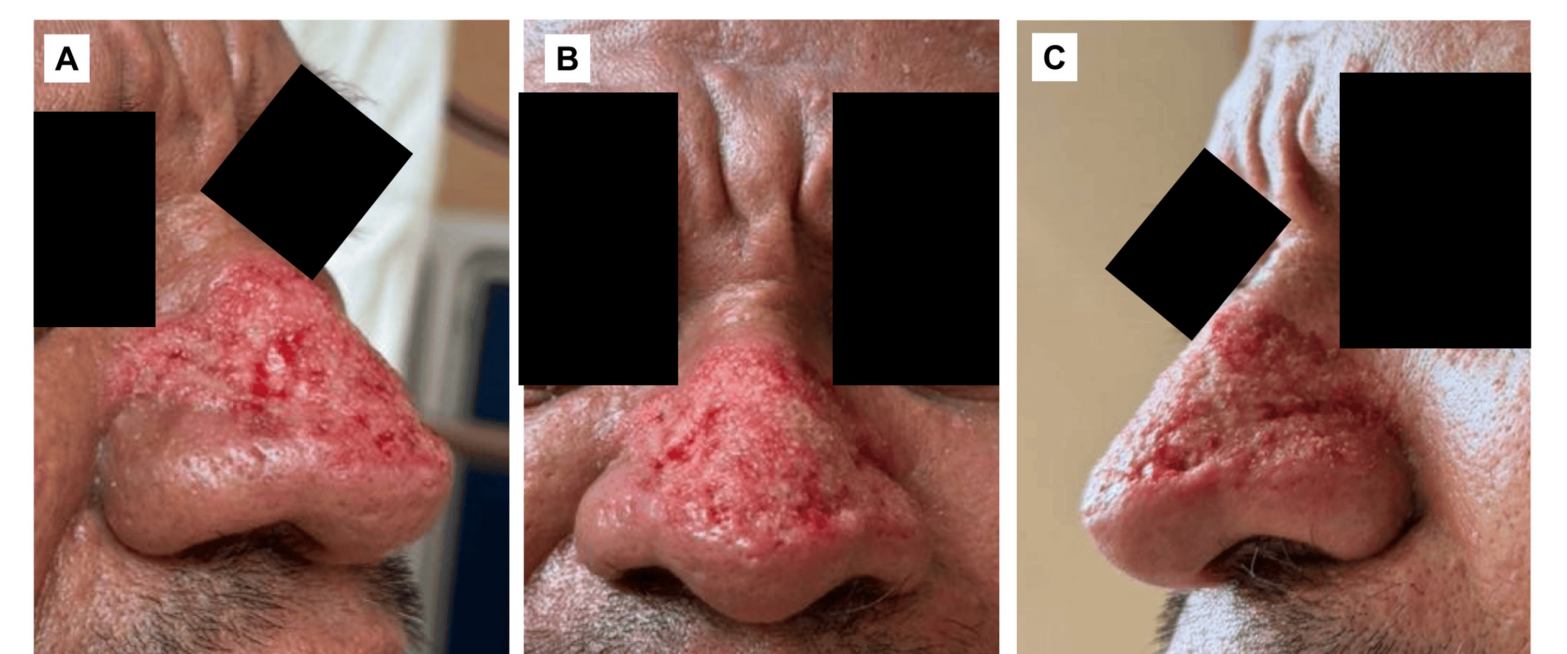
Immediate postoperative appearance following electrosurgical tangential decortication (A) Left lateral view: Left oblique view demonstrates controlled tangential debulking of hypertrophic nasal tissue with preservation of underlying dermal structures. (B) Frontal view: Frontal view shows marked reduction of lobulated nasal hypertrophy with uniform punctate bleeding consistent with appropriate depth of excision. (C) Right lateral view: Right oblique view illustrates restoration of the nasal contour without cartilage exposure or excessive thermal injury.

Sequential layer-by-layer excision was performed until the reticular dermis was reached. Particular attention was paid to preserving the deep pilosebaceous apparatus, respecting nasal aesthetic subunits, and avoiding over-resection that could expose cartilage. Uniform punctate bleeding served as the intraoperative indicator of appropriate depth. Final contour refinement was performed with controlled electrosurgical passes (Figures 2A-2C).

Results

**Figure 3:**
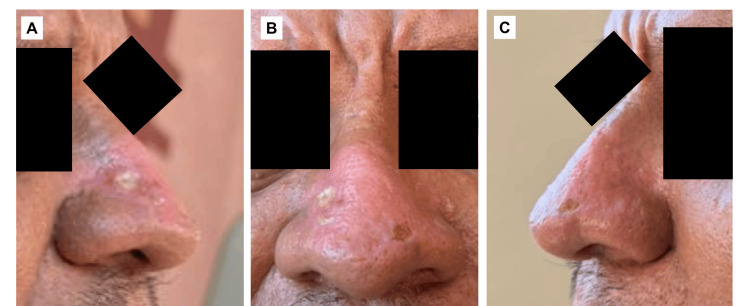
Follow-up on postoperative day 35 (A) Left lateral view: Symmetrical contour is restored with maintained nasal subunits. No hypertrophic scarring, keloid formation, or pigmentary alteration is evident. (B) Frontal view: Marked improvement is seen in the nasal contour with elimination of lobulated hypertrophy. Skin surface appears smooth with preserved adnexal architecture. Mild residual erythema is present, consistent with expected postoperative remodeling. (C) Right lateral view: Complete re-epithelialization is seen with restoration of physiologic nasal projection. No evidence of cartilage collapse, excessive tissue contraction, or alar distortion is observed.

Postoperative recovery was uneventful. Daily cleansing and topical antibiotic ointment were prescribed. No systemic antibiotics were required. Re-epithelialization progressed steadily without infection, delayed healing, or tissue necrosis. By postoperative day 35, complete epithelial coverage was achieved, nasal projection was physiologically restored, lobulated hypertrophy was eliminated, skin surface demonstrated smooth remodeling, no hypertrophic scarring or keloid formation occurred, and no pigmentary alteration was observed (Figures 3A-3C). The patient reported high satisfaction with aesthetic improvement and facial harmony.

## Discussion

Advanced rhinophyma represents a structural and fibrotic stage of rosacea in which medical therapy plays only an adjunctive role [6]. Progressive sebaceous gland hyperplasia and dermal fibrosis ultimately result in irreversible tissue hypertrophy requiring surgical intervention. The primary therapeutic objective is the removal of hypertrophic tissue while preserving sufficient adnexal structures to allow re-epithelialization and minimize scarring.

Multiple surgical techniques have been described, including scalpel excision, dermabrasion, cryotherapy, electrosurgery, and laser resurfacing [7-10]. Among these, CO₂ laser therapy has been widely reported due to precise vaporization and excellent hemostasis. Several studies have demonstrated favorable cosmetic outcomes with laser-based approaches, particularly in severe rhinophyma [8-10]. However, these techniques require specialized equipment and may be associated with prolonged postoperative erythema and pigmentary changes.

Electrosurgery provides an alternative approach that allows rapid tangential debulking with immediate hemostasis. The principal concern with electrosurgery is thermal spread and the potential for excessive tissue injury. Nevertheless, when performed in controlled layers and guided by punctate bleeding as a marker of dermal preservation, collateral damage is minimal. Preservation of the deep pilosebaceous units enables epithelial regeneration from adnexal remnants, reducing the risk of hypertrophic scarring and contracture [11,12].

In the present case, layered tangential excision was performed with careful preservation of nasal aesthetic subunits. Uniform punctate bleeding confirmed adequate depth of resection without cartilage exposure. Complete re-epithelialization occurred by postoperative day 35 with restoration of nasal contour and no pigmentary alteration. These findings are consistent with previously reported electrosurgical series demonstrating favorable cosmetic outcomes and low complication rates [5,7].

From a practical standpoint, electrosurgery offers several advantages, including low cost, wide availability, short operative time, and reproducibility. In many centers, particularly in resource-limited settings, access to CO₂ laser systems remains limited. Therefore, electrosurgical tangential decortication represents a scalable option for the management of advanced rhinophyma.

Limitations of this report include the relatively short follow-up period, lack of standardized morphometric measurements, absence of histopathologic confirmation, and non-standardized clinical photography conditions inherent to retrospective clinical documentation. Longer follow-up and larger prospective studies incorporating standardized outcome measures would be valuable to better assess long-term recurrence, scar evolution, and comparative efficacy relative to other surgical modalities.

Overall, this case supports the concept that surgical precision and depth control, rather than energy modality alone, are the primary determinants of outcome. When adnexal structures are preserved and nasal subunits respected, electrosurgery can achieve outcomes comparable to more technologically complex approaches.

## Conclusions

Electrosurgical tangential decortication proved to be a safe and effective treatment option for severe rhinophyma in this case. Controlled layer-by-layer excision with preservation of deep pilosebaceous structures allowed satisfactory re-epithelialization and restoration of the nasal contour at the 35-day follow-up.

Although this case demonstrated favorable early cosmetic outcomes without clinically evident complications, larger studies with longer follow-ups are needed to better evaluate recurrence risk, long-term scar evolution, and comparative outcomes relative to other surgical modalities. Electrosurgery may represent a practical alternative in appropriately selected patients, particularly in settings where access to laser-based technologies is limited.
